# Consensus of experts from the Spanish Pharmacogenetics and Pharmacogenomics Society and the Spanish Society of Medical Oncology for the genotyping of *DPYD* in cancer patients who are candidates for treatment with fluoropyrimidines

**DOI:** 10.1007/s12094-021-02708-4

**Published:** 2021-11-13

**Authors:** P. García-Alfonso, M. Saiz-Rodríguez, R. Mondéjar, J. Salazar, D. Páez, A. M. Borobia, M. J. Safont, I. García-García, R. Colomer, X. García-González, M. J. Herrero, L. A. López-Fernández, F. Abad-Santos

**Affiliations:** 1 Medical Oncology Department, Hospital General Universitario Gregorio Marañón, Sociedad Española de Oncología Médica (SEOM), C/Doctor Esquerdo, 46, 28007 Madrid, Spain; 2 Research Unit, Fundación Burgos por la Investigación de la Salud (FBIS), Hospital Universitario de Burgos, Sociedad Española de Farmacogenética y Farmacogenómica (SEFF), Burgos, Spain; 3 Medical Oncology Service, Hospital Universitario de la Princesa, Sociedad Española de Oncología Médica (SEOM), Madrid, Spain; 4 Research Institute of Hospital de la Santa Creu I Sant Pau, Sociedad Española de Farmacogenética y Farmacogenómica (SEFF), Barcelona, Spain; 5 Medical Oncology Department, Hospital de la Santa Creu I Sant Pau, Sociedad Española de Oncología Médica (SEOM), Barcelona, España; 6 Clinical Pharmacology Service, Hospital Universitario La Paz, Sociedad Española de Farmacogenética y Farmacogenómica (SEFF), Madrid, Spain; 7 Medical Oncology Service, Consorcio Hospital General Universitario de Valencia, Universidad de Valencia, CIBERONC, Sociedad Española de Oncología Médica (SEOM), Valencia, Spain; 8 Medical Oncology Service, Hospital Universitario de La Princesa y Cátedra de Medicina Personalizada de Precisión de la Universidad Autónoma de Madrid (UAM), Sociedad Española de Oncología Médica (SEOM), Madrid, Spain; 9 Hospital Pharmacy Service, Hospital General Universitario Gregorio Marañón, Sociedad Española de Farmacogenética y Farmacogenómica (SEFF), Madrid, Spain; 10 Pharmacogenetics Platform, IIS La Fe-Hospital La Fe and Pharmacology Department, Universidad de Valencia, Sociedad Española de Farmacogenética y Farmacogenómica (SEFF), Valencia, Spain; 11 Clinical Pharmacology Service, Hospital Universitario de La Princesa, Universidad Autónoma de Madrid, Sociedad Española de Farmacogenética y Farmacogenómica (SEFF), C/Diego de León, 62., 28006 Madrid, Spain

**Keywords:** 5-fluorouracil, Capecitabine, Dihydropyrimidine dehydrogenase, Genotypes, Pharmacogenetics, Toxicity

## Abstract

5-Fluorouracil (5-FU) and oral fluoropyrimidines, such as capecitabine, are widely used in the treatment of cancer, especially gastrointestinal tumors and breast cancer, but their administration can produce serious and even lethal toxicity. This toxicity is often related to the partial or complete deficiency of the dihydropyrimidine dehydrogenase (DPD) enzyme, which causes a reduction in clearance and a longer half-life of 5-FU. It is advisable to determine if a DPD deficiency exists before administering these drugs by genotyping *DPYD* gene polymorphisms. The objective of this consensus of experts, in which representatives from the Spanish Pharmacogenetics and Pharmacogenomics Society and the Spanish Society of Medical Oncology participated, is to establish clear recommendations for the implementation of genotype and/or phenotype testing for DPD deficiency in patients who are candidates to receive fluoropyrimidines. The genotyping of *DPYD* previous to treatment classifies individuals as normal, intermediate, or poor metabolizers. Normal metabolizers do not require changes in the initial dose, intermediate metabolizers should start treatment with fluoropyrimidines at doses reduced to 50%, and poor metabolizers are contraindicated for fluoropyrimidines.

## Introduction

Fluoropyrimidines or dihydropyrimidines (5-fluorouracil [5-FU], capecitabine, and tegafur) are antimetabolite drugs that are widely used to treat solid tumors, including breast and colorectal cancers and other gastrointestinal tract cancers. Each year, more than 2 million patients worldwide are diagnosed with a new cancer that is treated with fluoropyrimidines, mainly in combination with other antineoplastic drugs [1, 2]. Approximately 10–40% of patients treated with fluoropyrimidines develop severe toxicity (grade ≥ 3 on the *Common Terminology Criteria for Adverse Events* [CTCAE]), which may include myelosuppression, severe diarrhea, vomiting, stomatitis, mucositis, hand–foot syndrome (palmar–plantar erythrodysaesthesia), or neuropathy [3–6]. In 1% of patients, toxicity can be fatal [2]. It can occur in the first cycle of treatment, which suggests the importance of adjusting the initial dose of fluoropyrimidines for each patient before treatment starts [4].

The main enzyme responsible for eliminating fluoropyrimidines is dihydropyrimidine dehydrogenase (DPD), encoded by the *DPYD* gene. The partial or complete deficiency of this enzyme has been associated with greater toxicity from fluoropyrimidines [3, 6], since 5-FU accumulates, and more active metabolites are formed. The phenotype of DPD can be defined by the presence or absence of single-nucleotide polymorphisms (SNPs) in the *DPYD* gene that alter the activity of the DPD enzyme.

Several international guidelines recommend genotyping patients before giving them fluoropyrimidines [1, 2, 7], and dose adjustment reduces the risk of toxicity in subjects carrying *DPYD* mutations [6, 8, 9]. Currently in Spain, *DPYD* genotyping is not carried out in all patients who receive fluoropyrimidines. In May 2020, the Spanish Agency for Medicine and Health Products (AEMPS) published an informative note that recommended carrying out genotype and/or phenotype testing for DPD deficiency in patients who are candidates for dihydropyrimidines [10]. The objective of this consensus of experts, in which representatives of the Spanish Pharmacogenetics and Pharmacogenomics Society (SEFF) and the Spanish Society of Medical Oncology (SEOM) have participated, is to establish clear recommendations for the implementation of genotype and/or phenotype testing for DPD deficiency in patients who are candidates to receive dihydropyrimidines.

## Clinical use of fluoropyrimidines in cancer patients

### Indications and regimens of fluoropyrimidines

Fluoropyrimidines are essential in the treatment of multiple neoplasms, 5-FU and capecitabine being the most commonly used in solid tumors. The main indications for fluoropyrimidines [11, 12] are (1) colorectal cancer in the adjuvant and metastatic setting, both as monotherapy and in combination with oxaliplatin or irinotecan; (2) treatment of locally advanced rectal cancer in the neoadjuvant setting associated with radiotherapy; (3) treatment of localized anal canal cancer; (4) treatment of locally advanced or metastatic oesophageal cancer; (5) first-line treatment of metastatic gastric cancer in combination with a regimen that includes platinum, as well as of perioperative or adjuvant gastric cancer; (6) treatment of pancreatic cancer in the adjuvant or metastatic settings; (7) treatment of breast cancer in the adjuvant or metastatic settings; in the treatment of locally advanced or metastatic breast cancer, it can be administered as monotherapy after failure of taxanes and an anthracycline regimen or when subsequent treatment with anthracyclines is not indicated; and (8) treatment of inoperable, locally advanced, and recurrent or metastatic squamous-cell carcinoma of the head and neck.

5-FU is administered by intravenous injection as a bolus, perfusion, or continuous perfusion for 1–5 days, alone or modulated with leucovorin. Capecitabine is a prodrug of 5-FU that is administered orally at doses of 800–1250 mg/m^2^ body surface area every 12 h. Tegafur is infrequently used today, although it can be used at doses of 500–1000 mg/m^2^/day as part of various chemotherapeutic regimens. Fluoropyrimidines can be administered both as monotherapy and in combination with other antineoplastic agents and monoclonal antibodies. Table 1 summarizes the most commonly used chemotherapy regimens with fluoropyrimidines, classified by tumor type [13, 14].

**Table 1:** Chemotherapy regimens with the most widely used fluoropyrimidines by tumor type

Tumor	Scheme	Dose	Frequency (days)
Adjuvant colon	CAP	CAP 1.000–1.250 mg/m^2^/12 h, 14 days	Every 21
	CAPOX	CAP 850–1.000 mg/m^2^/12 h, 14 days OX 130 mg/m^2^, day 1	Every 21
	mFOLFOX-6	Leucovorin 400 mg/m^2^ 5-FU 400 mg/m^2^ bolus and 2.400 mg/m^2^ c.i. 46 h OX 85 mg/m^2^, day 1	Every 14
Neoadjuvant rectum	CAP and RT	CAP 825 mg/m^2^/12 h and RT Monday to Friday for 5 weeks	Daily
Metastatic colorectal*	CAPOX** and mFOLFOX-6	Same as in adjuvant	
	mFOLFIRI	Leucovorin 400 mg/m^2^ 5-FU 400 mg/m^2^ bolus and 2.400 mg/m^2^ c.i. 46 h Irinotecan 180 mg/m^2^, day 1	Every 14
Localized anal canal	5-FU, MIT-C and RT	5-FU 1.000 mg/m^2^/day c.i., days 1–4 MIT-C 10 mg/m^2^, day 1 × 2 cycles	Every 21
Localized oesophageal epidermoid	Cisplatin, 5-FU and RT	Cisplatin 75 mg/m^2^, day 1 5-FU 800 mg/m^2^/day c.i., days 1–5	Every 21
Perioperative gastric	FLOT	5-FU 2.600 mg/m^2^ c.i. 24 h Leucovorin 200 mg/m^2^ OX 85 mg/m^2^ Docetaxel 50 mg/m^2^, day 1	Every 14
Metastatic oesophageal and gastric	Cisplatin and CAP	Cisplatin 80 mg/m^2^, day 1 CAP 1.000 mg/m^2^/12 h, 14 days ± trastuzumab in HER2 + + +	Every 21
	EOX	Epirubicin 50 mg/m^2^ OX 130 mg/m^2^ CAP 625 mg/m^2^/12 h continuous	Every 21
	AL-SARRAF, TPF, XELOX and FOLFOX	Same as metastatic head and neck and adjuvant colon	
Pancreas	FOLFIRINOX	Leucovorin 400 mg/m^2^ 5-FU 400 mg/m^2^ bolus and 2.400 mg/m^2^ c.i. 46 h Irinotecan 150 mg/m^2^ OX 85 mg/m^2^, day 1	Every 14
Localized breast	FEC 100	5-FU 500 mg/m^2^ Epirubicin 100 mg/m^2^ Cyclophosphamide 600 mg/m^2^, day 1	Every 28
Metastatic breast	CAP and lapatinib	CAP 1.000 mg/m^2^/12 h, 14 days Lapatinib 1.250 mg/m^2^/day continuous	Every 21
	CMF	Cyclophosphamide 600 mg/m^2^ Methotrexate 40 mg/m^2^ 5-FU 600 mg/m^2^, day 1	Every 21
	TC	Docetaxel 75 mg/m^2^, day 1 CAP 1.250 mg/m^2^/12 h, 14 days	Every 21
	CAP and trastuzumab	CAP 1.000 mg /m^2^/12 h, 14 days Trastuzumab 6 mg/kg, day 1 (loading dose: 8 mg/kg)	Every 21
Neoadjuvant head and neck	TPF	Docetaxel 75 mg/m^2^ Cisplatin 75–100 mg/m^2^ 5-FU 1.000 mg/m^2^/day c.i., days 1–4, day 1	Every 21
Metastatic head and neck	EXTREME	Cisplatin 100 mg/m^2^, day 1 5-FU 1.000 mg/m^2^/day c.i., days 1–4 Cetuximab 250 mg/m^2^, days 1, 8 and 15 (loading dose: 400 mg/m^2^)	Every 21
	AL-SARRAF	Cisplatin 100 mg/m^2^ 5-FU 1.000 mg/m^2^/day c.i., days 1–5	Every 21

*5-FU* 5-fluorouracil, *AL-SARRAF* cisplatin and 5-fluorouracil, *CAP* capecitabine, *CAPOX* capecitabine and oxaliplatin, *CMF* cyclophosphamide, methotrexate and 5-fluorouracil, *EGFR* epidermal growth factor receptor, *EOX* epirubicin, oxaliplatin and capecitabine, *EXTREME* cisplatin, 5-fluorouracil and cetuximab, *FEC* 5-fluorouracil, epirubicin and cyclophosphamide, *FLOT* 5-fluorouracil, leucovorin, oxaliplatin and docetaxel, *FOLFIRI* leucovorin, 5-fluorouracil and irinotecan, *FOLFIRINOX* leucovorin, 5-fluorouracil, irinotecan and oxaliplatin, *FOLFOX* leucovorin, 5-fluorouracil and oxaliplatin, *c.i.* continuous infusion, *MIT-C* mitomycin C, *OX* oxaliplatin, *RT* radiotherapy, *TC* docetaxel and capecitabine, *TPF* docetaxel, cisplatin and 5-fluorouracil

*Associated with anti-VEGF (bevacizumab or aflibercept) or anti-EGFR (cetuximab or panitumumab) in patients with *RAS* wild type

**The use of capecitabine in combination with anti-EGFR is not approved

### Toxicity of fluoropyrimidines

In general, fluoropyrimidines are well-tolerated cytostatics with a manageable adverse event profile. However, the toxicity associated with these drugs is a recognized clinical problem that has significant consequences on the quality of life of patients. Approximately, 30% of those who receive 5-FU or capecitabine in monotherapy experience severe toxicity related to the treatment, which in 10–20% of cases may require hospitalization and is lethal in 0.5–1.0% of cases [15–17].

The toxicity profile of fluoropyrimidines varies depending on the administration regimen. With the most common regimen of 5-FU in continuous infusion, diarrhea and mucositis are the main dose-limiting problems, while myelosuppression and subacute palmar–plantar erythrodysaesthesia (hand–foot syndrome) are usually less frequent [15]. In contrast, an intravenous bolus of 5-FU causes more myelosuppression. With the usual oral regimen of capecitabine administration, the most frequent toxicities that lead to dose reduction or treatment interruption are hand–foot syndrome, diarrhea, and nausea [16]. Other common toxicities are fatigue, stomatitis, skin hyperpigmentation, photosensitivity, blepharitis, and epiphora. A rare but potentially serious adverse effect is cardiotoxicity associated with treatment with 5-FU or capecitabine. These are cases of angina secondary to coronary vasospasm that can cause myocardial damage, especially in patients with a history of cardiovascular disease. The angina usually appears at the beginning of treatment and is reversible within a few hours after stopping the administration of the drug. Neurological toxicities, such as neuropathy, cerebellar ataxia, or cognitive impairment, have also been described in fewer than 1% of patients [15, 16].

Advanced age brings a higher risk of serious adverse events, since it is correlated with more comorbidities, worse functional status, and lower creatinine clearance, so a reduction in the initial dose of 20–25% is recommended in the geriatric population and in patients with moderate or severe kidney failure (e.g., creatinine clearance of 30–50 ml/min estimated by Cockroft–Gault formula) [18]. If creatinine clearance is less than 30 ml/min, capecitabine is contraindicated. In patients with mild to moderate hepatic impairment, close monitoring of analytical parameters is recommended without specific dose adjustment recommendations [15–17].

### Pharmacokinetics, pharmacodynamics, and pharmacogenetics of fluoropyrimidines

**Fig. 1:**
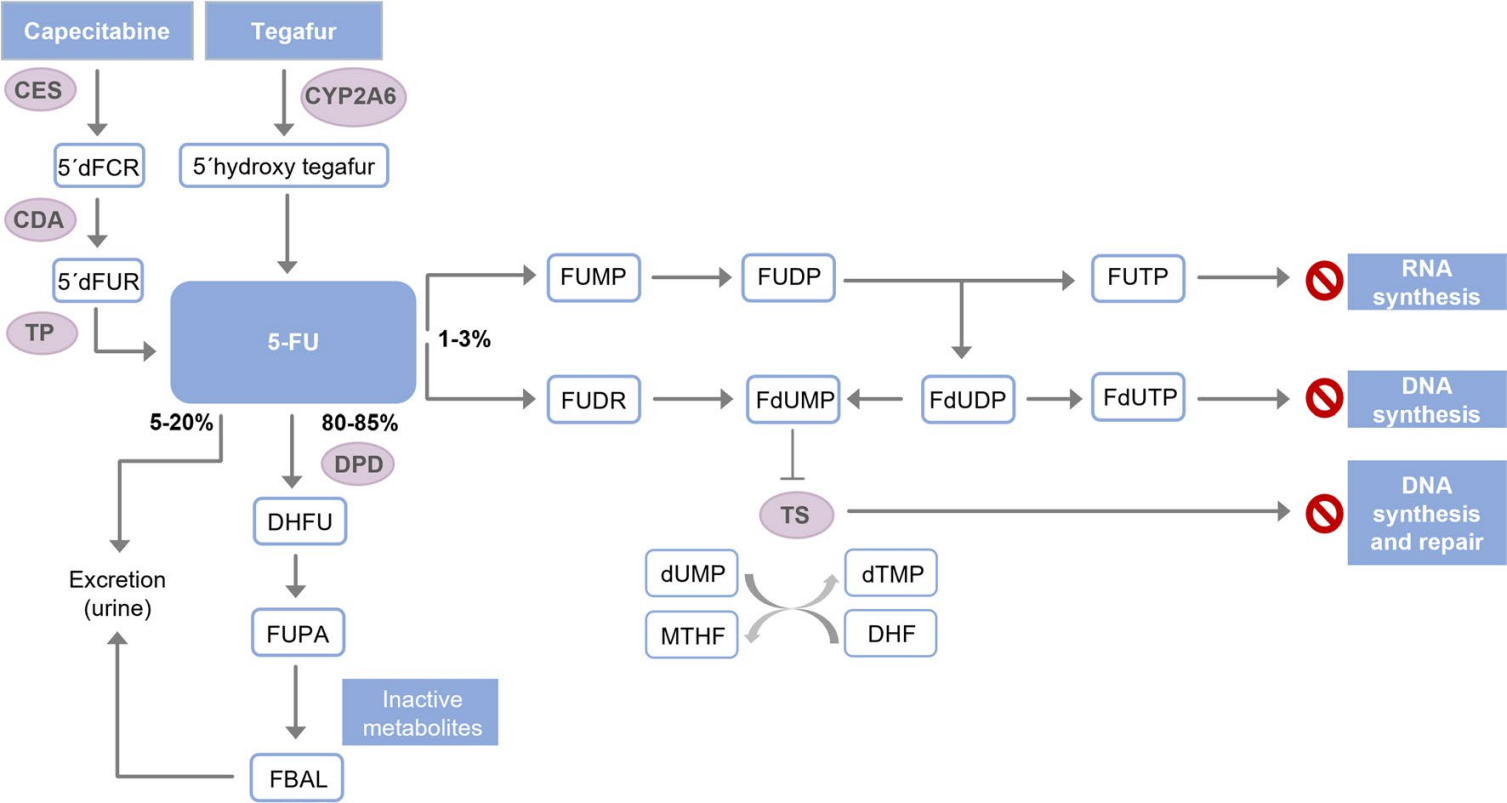
Metabolism and mechanism of action of fluoropyrimidines. *5-FU* 5-fluorouracil, *5′dFCR* 5′-deoxy-5-fluorocytidine, *5′dFUR* 5′-desoxy-5-fluorouridine, *CDA* cytidine deaminase, *CES* carboxylesterases, *CYP2A6* cytochrome P450 2a6, *DHF* dihydrofolate, *DHFU* dihydrofluorouracil, *DPD* dihydropyrimidine dehydrogenase, *dTMP* deoxythymidine monophosphate, *dUMP* deoxyuridine monophosphate, *FBAL* α -fluoro-β-alanine, *FdUDP* fluorodeoxyuridine diphosphate, *FdUMP* fluorodeoxyuridine monophosphate, *FdUTP* fluorodeoxyuridine triphosphate, *FUDP* fluorouridine diphosphate, *FUDR* fluorodeoxyuridine, *FUMP* fluorouridine monophosphate, *FUPA* 5-fluoro-ureidopropionic acid, *FUTP* fluorouridine triphosphate, *MTHF* 5,10-methylene tetrahydrofolate, *TP* thymidine phosphorylase, *TS* thymidylate synthase

5-FU is administered intravenously, while capecitabine and tegafur are prodrugs of 5-FU administered orally. 5-FU has no pharmacological activity but is converted into its active metabolites in the liver [19]. Only 1–3% of the administered dose of 5-FU is converted into cytotoxic metabolites that produce the antineoplastic effect (Fig. 1). More than 80% of 5-FU is converted into dihydrofluorouracil by the DPD enzyme and then into inactive metabolites [20, 21]. The rest of the 5-FU is excreted unmetabolized in the urine. Women show a lower clearance of 5-FU which can predispose them to a higher toxicity due to fluoropyrimidines, although the precise reason is unclear [22].

The main activation mechanism of 5-FU is conversion to fluorodeoxyuridine monophosphate (5-fluoro-2′-deoxyuridine 5′-monophosphate), which inhibits the enzyme thymidylate synthase, blocking the folate cycle and the synthesis of purines and pyrimidines, as described in Fig. 1 [21]. In addition, fluorouridine triphosphate and fluorodeoxyuridine triphosphate metabolites can be incorporated into RNA and DNA, respectively, which increases DNA repair mechanisms by base cleavage, leading to DNA fragmentation and cell death [21].

Capecitabine is absorbed in the intestinal wall and converted into 5′-deoxy-5-fluorocytidine and then into 5′-deoxy-5-fluorouridine (5′dFUR) by carboxylesterase and cytidine deaminase, respectively, both in the liver and tumor tissues [23]. 5′dFUR is converted into 5-FU by thymidine phosphorylase, which is generally more highly expressed in tumor tissues than in normal tissues. Tegafur is another prodrug of 5-FU, which is converted by the cytochrome P450 isoform CYP2A6 into an unstable intermediate metabolite, 5-hydroxytegafur, which decomposes spontaneously to form 5-FU [2] (Fig. 1).

### Description of the *DPYD* gene

The *DPYD* gene encodes the rate-limiting enzyme of the catabolism of fluoropyrimidines, DPD, and is approximately 950 kb long. It is located on chromosome 1p21.3 and consists of 23 exons [24, 25]. A multitude of genetic variants have been described that include polymorphisms and mutations that alter the protein sequence or RNA splicing (https://www.pharmvar.org/gene/DPYD) and that sometimes affect its enzymatic function. In this way, the high interindividual variability in the activity of DPD is mainly due to polymorphic variants in the *DPYD* gene. It is estimated that 0.01–0.5% of Caucasian individuals have a complete deficiency of the enzyme and 3–8% a partial deficiency. A deficiency in DPD activity is associated with a higher risk of severe toxicity due to fluoropyrimidine-based chemotherapy [26, 27] as a consequence of a lower rate of clearance and a longer half-life of 5-FU [28].

### Recommended *DPYD* gene variants to genotype

The AEMPS recommends genotyping for four *DPYD* variants in patients who are candidates for treatment with dihydropyrimidines (Table 2) [6, 10]: (1) DPYD*2A (rs3918290, c.1905 + 1G>A, IVS14 + 1G>A); (2) DPYD*13 (rs55886062, c.1679T>G, I560S); (3) *DPYD* c.2846A>T (rs67376798, D949V); and (4) *DPYD* c.1236G>A/HapB3 (rs56038477, E412E, in haplotype B3). These are variants of *DPYD* that lead to a loss of enzymatic function and are found with relatively higher frequency in the population. The DPYD*2A and DPYD*13 variants have the greatest impact on the activity of DPD, since it is no longer functional when homozygous; in heterozygous carriers, the reduction of activity is 50% and 68%, respectively, while the variants *DPYD* c.2846A>T and *DPYD* HapB3 cause a reduction of 30% and 35%, respectively, in heterozygous carriers [1].

**Table 2:** Characteristics of the recommended *DPYD* variants

Allele	Level of evidence assigned to allele*	Activity score	Level of evidence for dose titration^#^	Frequency in Europeans
No function				
DPYD*2A (rs3918290, c.1905 + 1G>A, IVS14 + 1G>A)	High	0	1A	1.0–1.2%
DPYD*13 (rs55886062, c.1679T>G, I560S)	Moderate	0	1A	0.1%
Decreased function				
*DPYD* c.2846A>T (rs67376798, D949V)	High	0,5	1A	0.8–1.4%
*DPYD* c.1236G>A/HapB3 (rs56038477, E412E, in haplotype B3)	High	0,5	1A	4.1–4.8%

*Based on *Clinical Pharmacogenetics Implementation Consortium* (CPIC) guidelines

^#^Based on clinical annotation levels of evidence of PharmGKB

DPYD*2A is located in the intronic region adjacent to exon 14 and causes an in-frame deletion of this entire exon, thus generating a non-functional protein [29]. The DPYD*13 and c.2846A>T variants are missense mutations that affect enzyme function [30, 31]. The *DPYD* c.1236G>A polymorphism is in perfect linkage disequilibrium with c.1129-5923C>G (rs75017182) (*r*^2^ = 1.0; *D*′ = 1.0) [32, 33]. Both define the HapB3 haplotype [5, 32, 33]. The *DPYD* c.1129-5923C>G (rs75017182) polymorphism in intron 10 produces a splicing error generating a protein with reduced activity [5, 32]. This SNP is probably the causal variant of the HapB3 haplotype [4].

In Europeans, the *DPYD* c.1129-5923C>G (HapB3) variant is the most common, with a frequency of 4.1–4.8% [1], followed by the DPYD*2A variants (1.0–1.2%) [1], *DPYD* c.2846A>T (0.8–1.4%) [1], and DPYD*13 (0.1%) [34]. Considering all the variants combined, approximately 7% of the European population carries at least one variant causing loss of function of the DPD enzyme [1]. Complete deficiency of DPD activity in carriers of two different variants or the same variant in homozygosity is very rare: It is estimated to occur in 0.01–0.50% of Caucasian individuals [10].

These four variants have been associated with a reduction in DPD activity and a statistically significant increase in the risk of toxicity [6]. The degree of evidence that supports the influence of each variant on DPD activity is different: for DPYD*2A, *DPYD* c.2846A>T, and HapB3, the evidence is strong (in vitro and clinical studies), while for DPYD*13 it is moderate (in vitro and *clinical/*ex vivo studies) [1].

To aid in the interpretation of the genotyping results and to define the phenotype of the patient, an individual activity score can be assigned to each of these four *DPYD* polymorphisms. Thus, if the alleles were *2A or *13 they would have a score of 0 and c.2846A>T and HapB3 of 0.5. If the allele is wild type, the score would be 1. To assign a global activity score, the sum of the individual scores of the two alleles is calculated. The optimal dose for the patient will depend on the activity calculated for DPD [1].

PharmGKB establishes different levels of evidence for pharmacogenetic associations with prescription recommendations [35]. For these four variants, the level of evidence is the highest: 1A. In fact, there are specific prescription guidelines available for these drug–variant pairs [36].

In the informative note published by AEMPS on May 11, 2020, it is noted that in addition to the four main variants mentioned above, there are rarer variants and other factors that may influence the development of toxicity associated with treatment with dihydropyrimidines [10]. Still, genotyping these four variants is the most well-established and simplest method [10].

### Determination of other variants of the *DPYD* gene

The four variants described in the previous section are not enough to cover all the activity deficits found for DPD. The gnomAD database currently includes 204 synonymous variants and 569 missense variants in *DPYD,* of which 40 have been related to the loss of enzymatic function [37]. The latest version of the *Clinical Pharmacogenetics Implementation Consortium* (CPIC) guide has a table of allelic functionality that includes 82 known variants, among which, in addition to the four mentioned in the previous section, 19 are considered to have no function and four to have diminished function [38]. However, most of these variants with phenotypic consequences are extremely rare and have not even been observed in studies with large cohorts [3, 39, 40]. In addition, in most cases, the activity assignment is based on in vitro or ex vivo studies, and the genotype–phenotype relationship has been corroborated in vivo only in a small proportion of cases [30, 41]. Besides the variant c.1905 + 1G>A (DPYD*2A), other mutations, such as c.2059-22T>G, c.321 + 1G>A, c.1740 + 2T, and c.2242 + 1G>T, produce a functional exon jump with the consequent loss of a part of the protein sequence and sometimes also a change in the open reading frame, generating a non-functional protein [42, 43]. However, the clinical evidence for the relationship of many of these variants with the toxicity of fluoropyrimidines is still limited, and there are no cost-effectiveness studies, which limits their wider incorporation into clinical practice. Among the known inactivating variants, in its guide to methodological recommendations and analytical interpretation, SEFF distinguishes six that it considers supported by a “moderate” level of evidence (Table 3) [44]. Typing these variants would be recommended in centers where the technique is easily accessible, to correctly assign the pharmacogenetic phenotypes of DPD despite their low frequency in the European population.

**Table 3:** Other very rare *DPYD* variants supported by a moderate level of evidence

SNP	cDNA variant	Protein variant	Impact on DPD activity	MAF European no Finnish (gnomAD)
Frameshift mutation				
rs72549303 (*3)	c.1898del	p.Pro633fs	Total loss of function	NR
rs72549309 (*7)	c.295_298TCAT	p.Phe100SerfsTer15	Total loss of function	0,0002016
Missense mutation				
rs1801266 (*8)	c.703C>T	p.Arg235Trp	Total loss of function	0,0000852
rs1801268 (*10)	c.2983G>T	p.Val995Phe	Total loss of function	NR
rs78060119 (*12)	c.1156G>T	p.Glu386Ter	Total loss of function	0,0000088
rs115232898	c.557A>G	p.Tyr186Cys	Decreased function	0,0000466

*MAF* minor allele frequency, *NR* not reported, *SNP* single-nucleotide polymorphism

*Identification of the allele

### Techniques to determine *DPYD* gene variants and accreditation of centers

Various techniques can be used to detect genetic variants of *DPYD* whose genotyping is recommended in this consensus of experts (i.e., DPYD*2A, *13, c.2846A>T, and HapB3) [44]. These are methods based on genotyping (selection of SNPs or arrays) or sequencing (Sanger or massive parallel sequencing). Another factor determining the choice of method is the number of genes and variants to be analyzed. Either the *DPYD* variants of interest alone could be typed, or they could be incorporated into a massive pharmacogenetic analysis that provides information on useful variants for other drugs. The fastest, simplest, and most economical way is to analyze the variants by real-time polymerase chain reaction [RT-PCR]). For this, any type of probe that discriminates a variant can be used, such as LightMix^®^ (Roche), TaqMan^®^ (Thermo Fisher), KASP^®^ (LGC Biosearch Technologies), or rhAmp^®^ (Integrated DNA Technologies) [45, 46]. A viable and economical alternative for laboratories that cannot do real-time PCR is to use conventional Sanger sequencing. Methods for the PCR amplification of each of the 23 exons of the *DPYD* gene have been described, so those that would amplify the positions of the four recommended variants can be selected [47]. Sequencing has the advantage that rare variants present in the sequenced regions can be detected. However, if the appropriate equipment is available, commercial kits such as the Elucigene *DPYD* LightMix in vitro diagnostics kit Multi-SNiP *DPYD* can be used to type the variants of interest.

As for the mass technologies useful for *DPYD* genotyping, custom or commercial SNP panels with thousands of SNPs in multiple genes, massive parallel sequencing, gene panels, exome sequencing, or genome sequencing can be used. All technologies are valid as long as at least the genetic variants recommended in this consensus of experts and its future updates are among those detected by each of these products and technologies.

Regardless of the technique used, it is essential that the laboratory has a recognized certification that supports the genotyping process. For this reason, SEFF launched in 2018 the first proficiency testing for accreditation of laboratories in pharmacogenetics (https://seff.es/grupo-proficiency-testing/). Since then, the second edition of this accreditation has been launched, which covers, among other polymorphisms of pharmacogenetic interest, the four minimum *DPYD* variants recommended for starting treatment with fluoropyrimidines. This accreditation, or a similar one, is necessary, regardless of the technique used. In addition, it is advisable that laboratories have other accreditations that certify the technique, the handling of samples, and the quality of the process, such as ISO 9001, ISO 14000 ISO 725, or ISO 15189. Any report of results must contain at least the following information regarding the genotyping: type of sample, technique used, and polymorphisms analyzed. It is also important to note in the report that the patient may carry rare variants that have not been analyzed but which may affect the phenotype assigned, and in the absence of any variants, it is assumed that the patient's phenotype would be categorized as a “normal” metabolizer.

## Individualized treatment with fluoropyrimidines based on genotyping of the DPYD gene

**Fig. 2:**
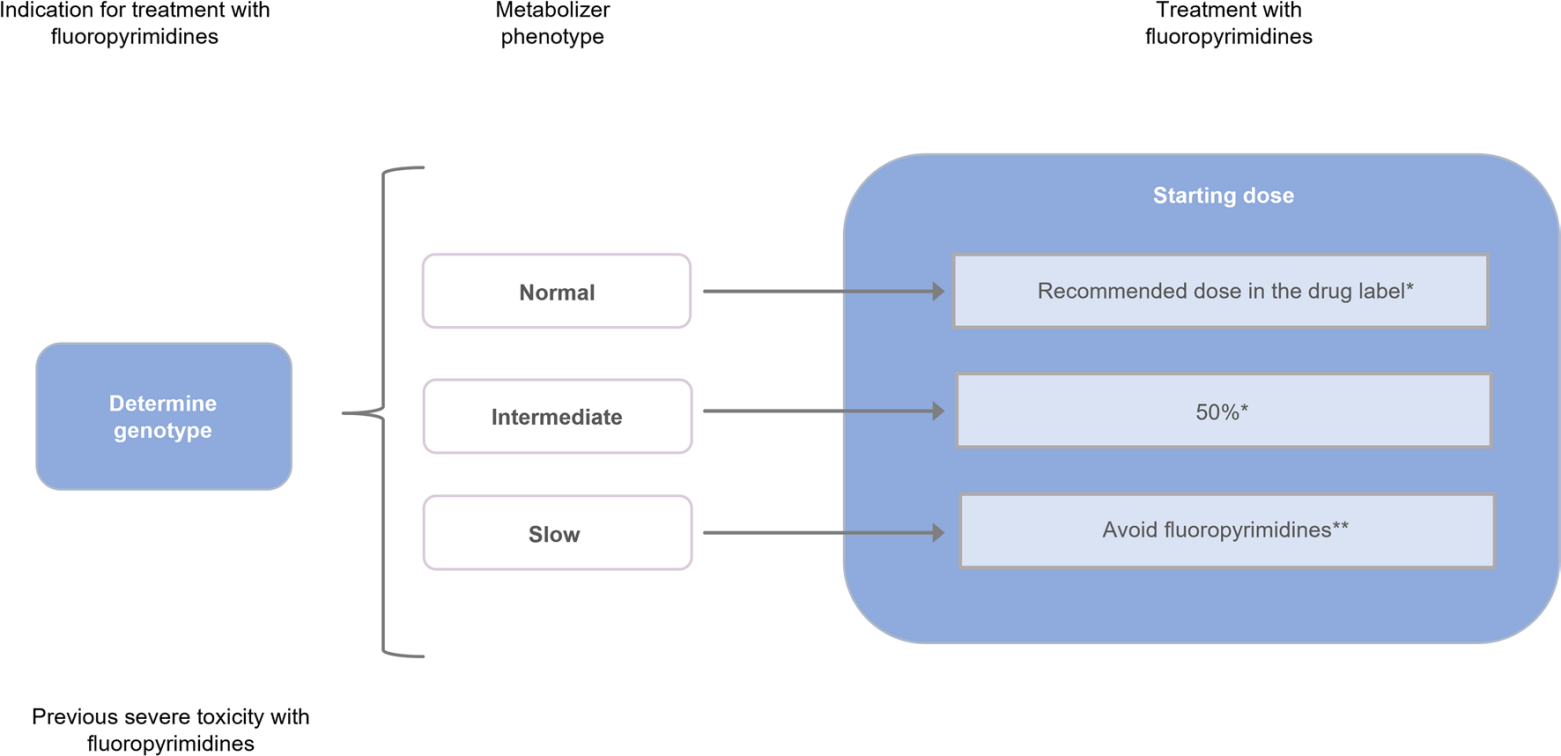
Decision-making algorithm in the administration of fluoropyrimidines in cancer patients. *Dose titration according to the toxicity observed. **Evaluate therapeutic alternatives

The current recommendations made by the Pharmacovigilance Risk Assessment Committee (PRAC) of the European Medicines Agency (EMA) to prevent any serious adverse events (particularly gastrointestinal, hematological, hand–foot syndrome, etc.) are based on assessing DPD activity in all patients who are candidates for receiving a fluoropyrimidine-based regimen, specifically the initial assessment of the following clinically validated mutations: c.1679T>G (DPYD*13, p.I560S), c.1905 + 1G>A (DPYD*2A), c.2846 A>T (p.D949 V), and c.1236 G>A (c.1129–5923 C>G, HapB3). Genotyping allows the classification of individuals as “normal” metabolizers (activity score of 2), “intermediate” metabolizers (activity score of 1–1.5), or “poor” metabolizers (activity score of 0–0.5) (Table 4). While the former does not require modifications in the initial dose, the intermediate group should start treatment with a dose reduced to approximately 50% and then escalate the dose in later cycles if no toxicity is observed. For poor metabolizers, the administration of fluoropyrimidines is contraindicated, and other therapeutic options should be considered (Fig. 2) [48, 49].

**Table 4:** Dosing of fluoropyrimidines according to DPD phenotype based on genotype

Phenotype	Genotype	Implications	Dosing recommendation
Normal metabolizer (Activity score 2)	Wild-type (absence of mutation)	Normal DPD activity and normal risk for fluoropyrimidine toxicity	According to the data sheet
Intermediate metabolizer (activity score 1–1.5)	Wild-type allele and mutated allele (*2A o *13 or c.2846A>T or HapB3)	Decreased DPD activity (30–70%) and increased risk for severe or even fatal drug toxicity when treated with fluoropyrimidines	Reduce starting dose by 50% followed by titration of dose based on toxicity or pharmacokinetics
	Two mutated alleles (c.2846A>T or HapB3)		
Poor metabolizer (Activity score 0–0.5)	Two mutated alleles (*2A or *13)	Complete or almost complete DPD deficiency and increased risk for severe or even fatal drug toxicity when treated with fluoropyrimidines	Contraindicated treatment with fluoropyrimidines; look for alternative agents*
	One mutated allele (*2A or *13) and one mutated allele (c.2846A>T or HapB3)		

*5-FU* 5-fluorouracil, *CPIC* Clinical Pharmacogenetics Implementation Consortium, *DPD* dihydropyrimidine dehydrogenase

*In the event that alternative agents are not considered a suitable therapeutic option and patient has an activity score of 0,5, CPIC indicates that 5-FU could be administered at a strongly reduced dose (< 25% of the normal dose) with early therapeutic drug monitoring of plasma concentration of 5-FU, to discontinue therapy if the drug level is too high [1]

The safety of individualized fluoropyrimidine treatment has been proven in several studies. In a prospective Dutch study, DPYD*2A, c.1679T>G, c.2846A>T, and c.1236G>A were genotyped in a series of more than 1,100 patients. It was found that prospective genotyping of *DPYD* is feasible in routine clinical practice and that dose reductions based on the results improved patient safety under fluoropyrimidine treatment. In that study, a reduction in the initial dose of 50% was adequate for DPYD*2A and c.1679T>G carriers, while a 25% dose reduction in c.1236G>A and c.2846A>T carriers was insufficient to reduce the risk of toxicity [6].

Another question is whether individualization can affect the efficacy of treatment. A cohort study compared the clinical evolution between carriers of the DPYD*2A variant treated with a 50% dose reduction and native paired controls who were treated with the full dose. The dose reduction did not seem to affect the overall survival of the patients (27 vs. 24 months; *p* = 0.47). However, the study was small, and there are no similar studies that have evaluated the clinical evolution of carriers of the other three variants [50].

A limitation of individualized treatment based on *DPYD* analysis is that the variants of *DPYD* that have been studied are predictive only in Western or northern European populations. In a recent Spanish study [47], 28 patients who presented severe toxicity induced by fluoropyrimidines and who were not carriers of the four recommended mutations were studied. They sequenced the full *DPYD* exome and phenotyped DPD by measuring uracilemia (U) and dihydrouracilemia (UH2) and the UH2/U ratio in the plasma. The DPYD*6 variant (c.2194G>A) was present in an unexpectedly high percentage (32%) of this population, and three of the patients studied did not have *DPYD* coding variants. Those findings suggested that other factors, such as other genes or epigenetic changes, may play important roles.

## Benefits obtained from the determination of *DPYD* gene variants

Dose adjustments based on the genotyping of *DPYD* reduce the risk of serious adverse reactions that can even lead to death. They can also lead to a decrease in the number of hospitalizations and associated costs. Genotyping *DPYD,* in addition to being a tool that reduces severe toxicity, is shown to be cost-effective.

A prospective, multicenter study conducted in the Netherlands analyzed the reduction in toxicity and total costs of treatment with fluoropyrimidines guided by the determination of DPYD*2A [51]. A total of 1,613 patients were genotyped before starting treatment with fluoropyrimidines, and the toxicity of the treatment guided by the DPYD*2A genotype was compared with the toxicity observed in historical controls (3,974 patients heterozygous for the DPYD*2A variant previously treated with a standard fluoropyrimidine dose) [51]. The risk of developing toxicity equal to or greater than grade 3 was reduced from 73% in historical controls to 28% in patients with guided treatment [51]. In addition, drug-induced death decreased from 10 to 0% [51]. The authors concluded that genotype*-*guided dosing of DPYD*2A allows appropriate systemic exposure to fluoropyrimidines and significantly improves treatment safety [51]. This study, published in 2015, had an important limitation: It only analyzed one of the main variants of the *DPYD* gene. Even so, although the difference was slight, early genotyping demonstrated cost savings when the average total cost of treatment per patient was evaluated, which was lower in patients with early genotyping (€ 2,772) than in those in the control group (€ 2,817) [51]. A later study of the same group in 1,103 patients, which analyzed the DPYD*2A, DPYD*13, and c.1236G>A variants, confirmed that the individualized dose of fluoropyrimidines guided by the *DPYD* genotype improved patient safety and reduced or had equivalent costs, but in no case involved additional cost [52].

A more comprehensive study conducted in 2018, which covered the variants DPYD*2A, DPYD*13, *DPYD* c.2846A>T, and DPYD*4, showed a greater benefit in terms of cost-effectiveness [53]. The costs associated with severe toxicity were evaluated after the start of chemotherapy with fluoropyrimidines and were compared with the costs of genotyping of all patients treated over 3 years (*N* = 134). Of these 134 patients, 23% (*N* = 30) developed toxicity, and of these, 17% (*N* = 5) had some mutation in *DPYD*. The authors calculated a total cost related to hospitalization for the toxicity of these five patients of € 232,061, with an average of € 46,412 per patient. If a cost of € 177 is assumed for each genotyping test, the cost of performing these tests prospectively on all 134 patients would have been € 23,718. Thus, early genotyping would have represented enormous savings, compared to the cost of hospital treatment of severe toxicity related to the chemotherapy in these patients [53].

In an Italian study [54] retrospective genotyping was carried out of the variants mentioned in the previous study plus the variant UGT1A1*28, in relation to irinotecan, in 550 patients. When comparing the costs related to toxicity and hospitalization in patients with any risk variant compared to patients with none, they concluded that the increase in cost per patient was € 2,975 in the group of patients with the risk variants.

Finally, a Spanish study on severe neutropenia caused by fluoropyrimidines concluded that, for prior genotyping to be cost-effective, it was sufficient to detect 2.21 patients with *DPYD* risk variants per 1,000 patients treated [45].

## Conclusions

5-FU or oral fluoropyrimidines are widely used in the treatment of cancer, especially gastrointestinal tumors and breast cancer. Their efficacy is widely recognized, both in monotherapy and in combination with other drugs; however, their administration produces severe toxicity in 10–40% of patients and even lethality in 0.5–1% [2]. Severe toxicity is particularly related to partial or complete deficiency of the DPD enzyme activity, which leads to a lower clearance rate and a longer half-life of 5-FU [3, 6]. It is estimated that between 0.01 and 0.5% of Caucasian individuals have a complete deficiency of the enzyme activity, and 3–8% have a partial deficiency. The high interindividual variability in the activity of DPD is mainly due to polymorphic variants in the *DPYD* gene [25].

Although 5-FU has been used for 60 years and knowledge of DPD activity dates back almost three decades, only recently has the genotyping of *DPYD* polymorphisms before using dihydropyrimidines in clinical practice been established. This consensus of experts, put together jointly by the SEOM and the SEFF, is intended to convey that, in the current era of personalized medicine, it is highly recommended to genotype *DPYD* polymorphisms before administering fluoropyrimidines. Following the recommendations made by the Pharmacovigilance Risk Assessment Committee of the EMA, it is advisable to screen for at least the following *DPYD* mutations that have clinically validated effects: (1) c.1679T>G (DPYD*13, p.I560S); (2) c.1905 + 1G>A (DPYD*2A); (3) c.2846 A>T (p.D949V); and (4) c.1236 G>A (c.1129–5923 C>G, HapB3) [10].

The genotyping of *DPYD* classifies individuals as normal, intermediate, or poor metabolizers. Normal metabolizers do not require changes in the initial dose, intermediate metabolizers should start treatment with fluoropyrimidines at doses reduced to 50%, and poor metabolizers are contraindicated for fluoropyrimidines, and other therapeutic options must be therefore considered.

The determination of the *DPYD* genotype before treatment with dihydropyrimidines offers advantages such as avoiding early toxicity that may be lethal and the deterioration of quality of life caused by toxicity. In addition, health care costs related to toxicity may be reduced, and it has been shown that *DPYD* genotyping is cost-effective. Even so, not all cases of severe toxicity are predictable by genotyping the four recommended variants. This highlights the need to expand the investigation to cover other variants of *DPYD* and other genes, to determine which techniques are the most appropriate for their genotyping, and which other factors may influence fluoropyrimidine toxicity.
